# Modulation of Serotonin-Related Genes by Extracellular Vesicles of the Probiotic *Escherichia coli* Nissle 1917 in the Interleukin-1β-Induced Inflammation Model of Intestinal Epithelial Cells

**DOI:** 10.3390/ijms25105338

**Published:** 2024-05-14

**Authors:** Yenifer Olivo-Martínez, Sergio Martínez-Ruiz, Cecilia Cordero-Alday, Manel Bosch, Josefa Badia, Laura Baldoma

**Affiliations:** 1 Departament de Bioquímica i Fisiologia, Facultat de Farmàcia i Ciències de l’Alimentació, Universitat de Barcelona, 08028 Barcelona, Spain; yeni_olivo@hotmail.com (Y.O.-M.); sergio_martinez_ruiz@ub.edu (S.M.-R.); corderocecilia16@gmail.com (C.C.-A.); 2 Biochemistry and Diseases Research Group, Facultad de Medicina, Universidad de Cartagena, Cartagena 130015, Colombia; 3 Institut de Biomedicina de la Universitat de Barcelona (IBUB), 08028 Barcelona, Spain; 4 Institut de Recerca Sant Joan de Déu (IRSJD), 08950 Barcelona, Spain; 5 Unitat de Microscòpia Òptica Avançada, Centres Científics i Tecnològics, Universitat de Barcelona, 08028 Barcelona, Spain; mbosch@ub.edu

**Keywords:** gut microbiota, postbiotics, extracellular vesicles, inflammatory bowel disease (IBD), intestinal barrier permeability, serotonin transporter (SERT), miR-24, miR-200a

## Abstract

Inflammatory bowel disease (IBD) is a chronic inflammatory condition involving dysregulated immune responses and imbalances in the gut microbiota in genetically susceptible individuals. Current therapies for IBD often have significant side-effects and limited success, prompting the search for novel therapeutic strategies. Microbiome-based approaches aim to restore the gut microbiota balance towards anti-inflammatory and mucosa-healing profiles. Extracellular vesicles (EVs) from beneficial gut microbes are emerging as potential postbiotics. Serotonin plays a crucial role in intestinal homeostasis, and its dysregulation is associated with IBD severity. Our study investigated the impact of EVs from the probiotic Nissle 1917 (EcN) and commensal *E. coli* on intestinal serotonin metabolism under inflammatory conditions using an IL-1β-induced inflammation model in Caco-2 cells. We found strain-specific effects. Specifically, EcN EVs reduced free serotonin levels by upregulating SERT expression through the downregulation of miR-24, miR-200a, TLR4, and NOD1. Additionally, EcN EVs mitigated IL-1β-induced changes in tight junction proteins and oxidative stress markers. These findings underscore the potential of postbiotic interventions as a therapeutic approach for IBD and related pathologies, with EcN EVs exhibiting promise in modulating serotonin metabolism and preserving intestinal barrier integrity. This study is the first to demonstrate the regulation of miR-24 and miR-200a by probiotic-derived EVs.

## 1. Introduction

The gut microbiota plays crucial roles in the physiology of the gastrointestinal tract. This microbial community assists in digestion, provides competitive mechanisms against enteric pathogens, and contributes to the development of the host immune system and maintenance of the intestinal epithelial barrier [1]. Microbiota–host interaction takes place at the complex interface of the intestinal mucosa, which includes several components that comprise the intestinal epithelial barrier. The mucus layer that covers the intestinal epithelium is formed by the extracellular glycoprotein mucin-2 (MUC-2) and contains antimicrobial peptides and Immunoglobulin A secreted by epithelial and immune cells, respectively. This layer creates a physical and chemical barrier that prevents luminal bacteria from reaching the epithelial cells [2]. Below the mucus layer, the intestinal epithelium is formed by a monolayer of epithelial cells sealed by tight junctions (TJs). These junctions work together to protect the host against harmful molecules and infectious agents by forming a selectively permeable barrier. Intestinal epithelial cells also play a fundamental role in sensing gut microbes through specific immune receptors, namely pattern recognition receptors (PRRs), which recognize conserved microbial-associated molecular patterns (MAMPs). In response to gut microbes, intestinal epithelial cells secrete immune mediators that modulate host immune responses, sustaining a well-balanced relationship between the gut microbiota and the host immune system [3]. Due to the anatomical structure of the gut barrier, microbiota–host interactions do not involve direct cell-to-cell contacts. Instead, they are principally mediated by microbiota-secreted factors, such as metabolites and extracellular vesicles (EVs) [4]. 

The proper physiology and homeostasis of the host depend on the integrity of the epithelial barrier. Factors that disturb the intestinal environment trigger imbalances in the gut microbiota’s composition and diversity (dysbiosis). This condition negatively affects the intestinal barrier and host-balanced responses. Disruption of the epithelial barrier and the subsequent increase in paracellular permeability allow for the translocation of intestinal microbes and their components into the lamina propria. The bacterial molecules activate the host immune system, triggering inflammatory responses that cause the dysfunction in many organs and tissues. High levels of circulating pro-inflammatory mediators and microbial products negatively disrupt the equilibrium of normal communication networks between the gut and distal organs, thus contributing to the development of a wide variety of intestinal and extraintestinal diseases [5,6,7]. The gut microbiota modulates the intestinal barrier through several mechanisms and factors. Besides the well-known modulation through immune receptors, the microbiota greatly influences other receptors/signaling pathways that control intestinal permeability, such as those activated by serotonin, endocannabinoids, or bile salts [8,9]. 

Serotonin, also known as 5-hydroxytryptamine (5-HT), is a key mediator of intestinal function. In this tissue, it modulates the enteric nervous system, immune responses, inflammation, gut motility, and barrier integrity [10]. Importantly, about 95% of the body’s serotonin is produced in the gut by enterochromaffin cells (ECCs), a population of intestinal epithelial cells that establish synapses with vagal neurons. Dietary tryptophan is the precursor for intestinal 5-HT synthesis in response to many stimuli, including signals from the gut microbiota. In ECCs, tryptophan is converted into 5-hydroxytryptophan through the action of tryptophan hydroxylase 1 (TPH1) and subsequently transformed into serotonin through a decarboxylase reaction. Free intestinal serotonin levels depend on the serotonin transporter (SERT, encoded by *slc6a4*), located at the apical and the basolateral sides of the cell membrane. Once released, serotonin can be taken up by intestinal epithelial cells through SERT and further inactivated by monoamine oxidase, the first enzyme of the serotonin degradation pathway. In addition, 5-HT released from the basolateral side can interact with specific receptors (5-HTR) of the surrounding epithelial, immune, and neuronal cells, triggering transduction signals that ultimately control vagal reflexes, gut motility, and barrier permeability. However, most of the intestinal 5-HT is distributed through the bloodstream, acting as an endocrine regulatory signal for the whole body [10]. Importantly, the gut microbiota modulates the expression of host serotonergic genes, thus influencing serotonin bioavailability and actions [11,12,13]. In this context, activation of the immune receptors toll-like receptor 2 (TLR2), toll-like receptor 4 (TLR4), and nucleotide-binding oligomerization domain protein-1 (NOD1) by microbial ligands inhibits SERT activity and/or expression in intestinal epithelial cell lines and animal models [14,15,16]. In addition, some microbiota species can synthesize serotonin and other tryptophan-derived metabolites, such as indole derivatives. Microbial tryptophan metabolites act through the aryl hydrocarbon intracellular receptor (AHR) and protect the gut barrier’s integrity [17]. 

Disruption of serotonin homeostasis in the gut, characterized by an excess level of extracellular serotonin, can lead to intestinal inflammation and leaky gut, thereby contributing to pathological disorders, such as inflammatory bowel disease (IBD) or irritable bowel syndrome (IBS) [10,18,19]. In the intestinal epithelium of Crohn’s disease (CD) and ulcerative colitis (UC) patients, elevated levels of serotonin correlated with reduced expression of SERT [20] and upregulation of miRNAs that target SERT mRNA, such as miR-24 and miR-200a [21,22]. Also, serotonin signaling modulates the gut microbiota composition towards a dysbiosis profile that causes inflammation and, therefore, susceptibility to colitis [23]. 

The interconnection between intestinal dysbiosis, serotonin, barrier disruption, and exacerbated immune responses provides the basis for the design of microbiome-oriented therapeutic strategies to restore intestinal homeostasis in IBD patients. One such strategy is the manipulation of the gut microbiota composition and function through probiotic-based interventions. Clinical trials have shown that the probiotic *Escherichia coli* Nissle 1917 EcN (EcN) shows comparable efficacy to the commonly used drug mesalazine in ameliorating clinical symptoms [24]. At present, the identification of novel probiotic strains for IBD treatment is an emerging area. Among them, *Akkermansia muciniphila* and *Faecalibacterium prausnitzii* are considered promising candidates for next-generation probiotics [25,26]. Importantly, the probiotics EcN, *A. muciniphila,* and *F. prausnitzii* improve intestinal serotonin balance by modulating serotonergic genes [11,12,13]. 

The administration of viable probiotics entails certain concerns, particularly in individuals with a weakened immune system. In these cases, probiotic treatment could enhance inflammatory responses and turn harmless probiotic bacteria into detrimental microorganisms. To avoid safety risks, upcoming microbiome-based therapies to target the gut involve postbiotics [26]. According to the recent definition by the International Scientific Association for Probiotics and Prebiotics (ISAPP), the term postbiotic refers to a preparation of inanimate (non-replicative) microorganisms or their components that confer a health benefit on the host, including bacterial cell components, biomolecules, metabolites, and secreted bioactive compounds [27]. In this context, probiotic/microbiota EVs are emerging as potential novel postbiotics [28,29]. Bacterial EVs are nano-scale bilayer structures originating from bacterial membranes that enclose biological components produced by the parental strain, including MAMPs, proteins, metabolites, DNA, and RNA. Recent research has provided scientific evidence that EVs mediate microbiota functions by transporting and delivering effector molecules into host cells. These molecules modulate host signaling pathways and cellular processes, including immune and defense responses. The effects exerted by microbiota EVs are strain-specific as they depend on their bacterial origin and cargo [4]. In this context, our studies have shown that EVs from probiotic and commensal *E. coli* strains modulate epithelial barrier function [30,31] and activate regulatory mechanisms to keep balanced anti-/pro-inflammatory responses [32,33]. Microbiota EVs stimulate dendritic cells to drive specific T-cell responses through several mechanisms that include activation of TLR–signaling pathways, regulation of miRNA expression, and differential release of immune mediators through exosomes [34,35]. Moreover, oral gavage of EVs from the probiotic EcN ameliorated colitis progression in dextran-sodium-sulphate-treated mice [36]. Recent reports show that EVs from *A. muciniphila* and *F. prausnitzii* modulate the expression of genes encoding enzymes and receptors of the serotonin system [12,13].

Given that the gastrointestinal tract is a major pool of serotonin in humans and the intestinal microbiome modulates the serotonin system, postbiotic-based interventions are foreseen as a therapeutic strategy to manipulate serotonergic signaling in IBD and other pathologies associated with exacerbated intestinal serotonin levels [37,38]. In this study, we aimed to evaluate whether EVs isolated from intestinal *E. coli* strains, specifically the probiotic EcN and the commensal EcoR12, could modulate intestinal serotonin metabolism under inflammatory conditions. We used the Caco-2 in vitro cellular model to induce inflammation with interleukin-1β (IL-1β), a major pro-inflammatory cytokine produced in IBD [39]. The anti-inflammatory effects of the bacterial EVs were assessed by analyzing the expression of genes and proteins of the serotonin system, pro-inflammatory cytokines, oxidative stress-related enzymes, and tight junction proteins. To deepen knowledge on the mechanisms underlying regulation of the serotonergic system, expression analysis of SERT-controlling miRNAs was approached. All assays were also performed in Caco-2 cell monolayers in the absence of IL-1β to elucidate how the regulatory mechanisms function under both physiological and pathological conditions. 

## 2. Results

### 2.1. Modulation of the Serotonergic System by EcN or EcoR12 EVs

The effect of EVs on the serotonergic system was evaluated in Caco-2 cells following two settings: (i) polarized cell monolayers as a model of the intact intestinal epithelium and (ii) polarized cell monolayers treated with IL-1β as an inflammation model of increased intestinal permeability. 

Before conducting the analysis, we established the inflammation model conditions. Initially, we assessed the impact of IL-1β concentration on Caco-2 cell viability following 48 h of incubation. The MTT results revealed that IL-1β concentrations of 1 or 10 ng/mL did not cause any cytotoxic effect because cell viability was close to 100% (Figure S1A). When IL-1β was added at 100 ng/mL, cell viability tended to decrease to approximately 80%. However, differences versus control cells were not statistically significant. Next, we determined the concentration of EVs to be used in the experiments. For this purpose, Caco-2 cell monolayers were exposed to EVs derived from EcN or EcoR12 at two different doses, 10 μg/mL and 60 μg/mL, for 48 h. The expression of the serotonergic genes *TPH1* (encoding tryptophan hydroxylase-1), *SERT* (encoding serotonin reuptake transporter), and *MAO* (encoding monoamine oxidase) was analyzed through RT-qPCR (Figure S1B). At the lowest concentration of EVs (10 μg/mL), no significant differences in the mRNA levels of these genes were observed compared to untreated control cells, except for *TPH1*, which was upregulated through treatment with EcN EVs (*p* < 0.05). Increasing the concentration of EcN EVs to 60 μg/mL resulted in higher *TPH1* mRNA levels (*p* ≤ 0.01). Regarding *SERT* expression, upregulation of this gene was only observed at the highest concentration (60 μg/mL) of both EcN and EcoR12 EVs (*p* < 0.05 vs. control). In contrast, *MAO* mRNA expression was not influenced by treatment with EcN EVs or EcoR12 EVs at any concentration tested. 

Based on these results and the existing literature [40], we established the experimental conditions for the inflammation model as follows. Polarized Caco-2 cells were subjected to a 3 h pretreatment with EVs (60 µg/mL) derived from either the probiotic EcN or the commensal EcoR12 followed by exposure to IL-1β (10 ng/mL) for an additional 48 h. The EVs were kept in the culture medium until the end of the experiment. In the model of an intact intestinal epithelium, Caco-2 cells were treated with EVs (60 µg/mL) for 48 h in the absence of IL-1β. 

**Figure 1:**
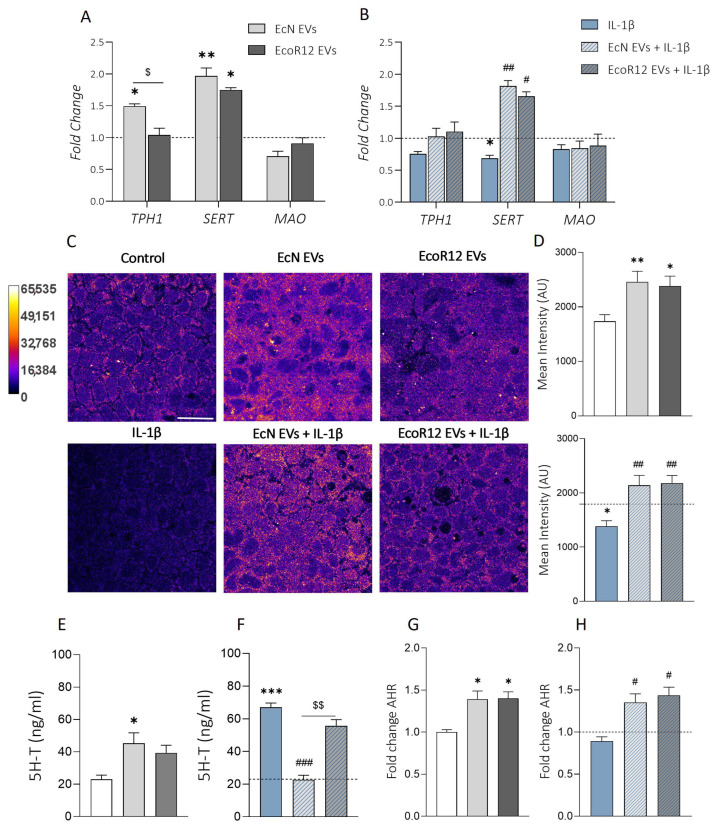
Modulation of the serotonergic system by EVs from the probiotic EcN and the commensal EcoR12 in Caco-2 cells. (**A**,**B**) mRNA expression levels of *SERT*, *TPH1,* and *MAO* in Caco-2 cell monolayers. Relative mRNA levels were measured through RT-qPCR using GAPDH as the reference gene. (**A**) Caco-2 cells were challenged with EVs (60 µg/mL) from EcN or EcoR12 for 48 h (intact intestinal epithelial model). (**B**) Caco-2 cells were pretreated with EVs (60 µg/mL) from EcN or EcoR12 for 3 h and then challenged with IL-1β (10 ng/mL) for 48 h (intestinal inflammation model). (**C**,**D**) Quantification of SERT through immunofluorescence confocal microscopy. (**C**) Representative confocal maximal projection images are shown. Images are color-coded with the Fire look-up table, and its calibration bar is shown on the left. Scale bar, 20 µm. Consistent with the intracellular SERT trafficking through membranous structures, the fluorescent signal is distributed in the cytoplasm. (**D**) Quantification of the SERT mean intensity in Caco-2 cells after the indicated treatments. Data are presented as mean ± SEM of arbitrary intensity units (AU) (*n* = 3 independent biological replicates). (**E**,**F**) Secreted 5-HT levels were quantified through ELISA in culture supernatant after 48 h of incubation following the model of the intact intestinal epithelium (**E**) or the inflammation model (**F**). (**G**,**H**) Relative mRNA levels of *AHR* in the model of the intact intestinal epithelium (**G**) or under conditions of IL-1β-induced inflammation (**H**). In all panels, data are expressed as mean ± SEM from 3 independent experiments, and control values were indicated by bars or dashed lines. Differences were evaluated with one-way ANOVA, followed by post hoc Tukey’s. * *p* ≤ 0.05, ** *p* ≤ 0.01, *** *p* ≤ 0.001 vs. control (white bar or dashed line); ^#^ *p* ≤ 0.05, ^##^ *p* ≤ 0.01, ^###^ *p* ≤ 0.001 vs. IL-1β group; ^$^ *p* ≤ 0.05, ^$$^ *p* ≤ 0.01, significance between cells stimulated with EcN and EcoR12 EVs.

In the model mimicking an intact intestinal epithelium, the results from the RT-qPCR analysis showed differential patterns of gene expression depending on the treatment (Figure 1A). As stated above, EcN EVs significantly upregulated *TPH1* (*p* ≤ 0.05 vs. control), whereas EcoR12 EVs did not modify its expression, resulting in *TPH1* mRNA levels that were indistinguishable from those of untreated control cells. Both EcN EVs (*p* ≤ 0.01) and EcoR12 EVs (*p* ≤ 0.05) triggered *SERT* upregulation. Under IL-1β-induced inflammation conditions (Figure 1B), treatment with this cytokine significantly decreased *SERT* expression (*p* ≤ 0.05 vs. control). Remarkably, *SERT* downregulation was counteracted by EcN and EcoR12 EVs, leading to *SERT* mRNA levels that were higher than those of untreated control cells. The induction of *TPH1* expression by EcN EVs was not evident under IL-1β-induced inflammation conditions. 

To confirm the impact of EcN and EcoR12 EVs on *SERT* expression, we carried out immunofluorescence staining followed by confocal laser scanning microscopy to quantify SERT protein levels in Caco-2 cell monolayers challenged under the established experimental conditions. Representative images are presented in Figure 1C. The SERT signal was quantified as described in the Material and Methods section (data collected from three independent biological experiments with five different fields of view with 30–40 cells each). Consistent with the mRNA expression profiles, the upregulation of SERT in cells incubated with EVs was accompanied by an increase in the signal intensity (Figure 1D). This correlation was observed in the model of the intact intestinal epithelium (*p* ≤ 0.01 for EcN EVs, *p* ≤ 0.05 for EcoR12 EVs vs. control) and in the inflammation model induced by IL-1β (*p* ≤ 0.01 for both EcN and EcoR12 EVs vs. IL-1β treated cells). 

To assess the impact of SERT modulation by microbiota/probiotic EVs on serotonin levels, 5-HT was quantified through ELISA in the culture supernatants. In the model of the intact intestinal epithelium, 5-HT secreted levels were increased through treatment with EcN EVs (*p* ≤ 0.05) or EcoR12 EVs compared to control cells (Figure 1E). However, under conditions of IL-1β-induced inflammation, the results showed differential regulation by EcN and EcoR12 EVs. Treatment with IL-1β resulted in a three-fold increase in secreted 5-HT levels compared to control cells (*p* ≤ 0.001). The IL-1β-induced increase in 5-HT secretion was impaired by EcN EVs (*p* < 0.01 vs. IL-1β), whereas EcoR12 EVs did not exhibit any modulatory effect on 5-HT availability (Figure 1F). 

In addition to its conversion into serotonin by host metabolism, dietary tryptophan is metabolized to different bioactive compounds through microbial enzymes. These microbial metabolites interact with the intracellular receptor AHR of epithelial cells to modulate several pathways, including intestinal inflammation [10]. In this context, we sought to analyze the regulation of the *AHR* gene by EcN or EcoR12 EVs in our experimental model. EVs from both gut-beneficial strains upregulated *AHR* in all conditions tested (*p* ≤ 0.05), without significant differences between strains (Figure 1G,H).

### 2.2. Regulation of miRNAs That Target SERT mRNA by EcN or EcoR12 EVs

The results presented above suggested a complex regulatory network governing serotonin availability in response to microbiota/probiotic EVs in the context of intestinal inflammation. To examine these regulatory mechanisms, the expression of miRNAs known to post-transcriptionally regulate SERT expression, namely miR-24 and miR-200a [41,42], was evaluated.

**Figure 2:**
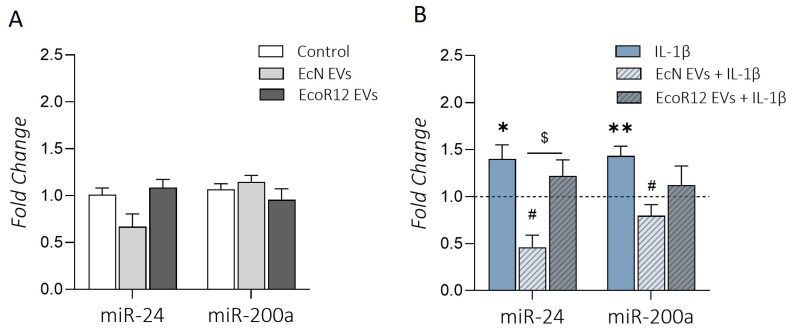
Regulation of miR-24 and miR-200a by EcN or ECOR12 EVs. (**A**) Intact intestinal epithelium model. Caco-2 cells were challenged with EVs (60 µg/mL) from EcN or EcoR12 for 48 h. (**B**) Inflammation model. Caco-2 cells were treated with EVs (60 µg/mL) from EcN or EcoR12 for 3 h and then challenged with IL-1β (10 ng/mL) for 48 h. Relative expression levels of the indicated miRNAs were measured through RT-qPCR and normalized to the U6 reference gene. In all panels, data are expressed as mean ± SEM from three independent experiments. Differences were evaluated with one-way ANOVA, followed by post hoc Tukey’s. * *p* ≤ 0.05, ** *p* ≤ 0.01 vs. control cells (bars in panel (**A**) or dashed line in panel (**B**)); ^#^ *p* ≤ 0.05 vs. IL-1β-treated cells; ^$^ *p* ≤ 0.05, significance between cells stimulated with EcN and EcoR12 EVs.

Under conditions of an intact intestinal epithelium, no differences in the relative levels of miR-24 and miR-200a were observed between control and EV-treated cells (Figure 2A). In contrast, under conditions of IL-1β-induced inflammation, the RT-qPCR results revealed distinctive miRNA expression patterns. When compared to the control, exposure to IL-1β induced a significant upregulation of miR-24 (*p* < 0.01) and miR-200a (*p* < 0.05) (Figure 2B). Remarkably, incubation with EcN EVs caused a significant decrease in the expression of both miRNAs compared to IL-1β-treated cells in the absence of EVs (*p* < 0.05). In contrast, stimulation with EcoR12 EVs was unable to prevent the IL-1β-induced-upregulation of both miRNAs (Figure 2B). These results revealed an inverse correlation between the expression levels of the examined miRNAs and *SERT* in Caco-2 cells treated with IL-1β. This finding is consistent with the fact that upregulation of miR-24 and miR-200a during intestinal inflammation suppresses *SERT* mRNA and protein expression. In turn, SERT suppression is associated with increased 5-HT availability, as assessed through ELISA in the supernatant of Caco-2 cells treated with IL-1β (Figure 1F). In the IL-1β model, the upregulation of *SERT* in cells treated with EVs from the probiotic EcN correlated well with the downregulation of both miRNAs. However, this inverse association was not observed in cells treated with EcoR12 EVs, thus suggesting that EVs from this commensal may regulate *SERT* expression through other mechanisms.

### 2.3. Modulation of Innate Immune Receptors by EcN or EcoR12 EVs 

**Figure 3:**
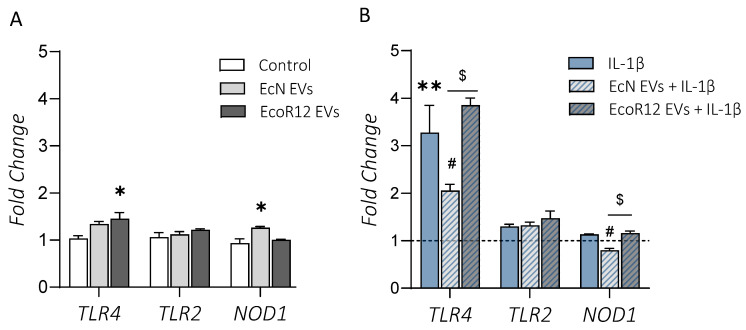
Modulation of TLR2, TLR4, and NOD1 gene expression by EcN or EcoR12 EVs. (**A**) Intact intestinal epithelium model. Caco-2 cells were challenged with EVs (60 µg/mL) from EcN or EcoR12 for 48 h. (**B**) Inflammation model. Caco-2 cells were treated with EVs (60 µg/mL) from EcN or EcoR12 for 3 h and then challenged with IL-1β (10 ng/mL) for 48 h. Relative mRNA levels of the indicated genes were measured through RT-qPCR using GAPDH as the reference gene. In all panels, data are expressed as mean ± SEM from three independent experiments. Differences were evaluated with one-way ANOVA, followed by post hoc Tukey’s. * *p* ≤ 0.05, ** *p* ≤ 0.01 vs. control cells (bars in panel (**A**) or dashed line in panel (**B**)); ^#^ *p* ≤ 0.05 vs. IL-1β-treated cells; ^$^ *p* ≤ 0.05, significance between cells stimulated with EcN and EcoR12 EVs.

The interplay between microbiota signals and immune receptors has a great influence on the intestinal serotonin levels and inflammation [19]. To decipher the mechanisms underlying the differential modulation of the serotoninergic system by EcN and EcoR12 EVs under inflammatory conditions, we analyzed their impact on the expression of genes encoding TLR2, TLR4, and NOD1 in polarized Caco-2 cell monolayers. In the model of the intact intestinal epithelium, EcoR12 EVs promoted upregulation of *TLR4*, whereas EcN EVs triggered upregulation of *NOD1* compared to untreated control cells (Figure 3A). In both cases, the increase in the mRNA levels of these genes, although statistically significant (*p* ≤ 0.05), was lower than 1.5-fold. In the intestinal inflammation model, the exposure to IL-1β significantly increased *TLR4* mRNA levels by about 3.5-fold (*p* < 0.01) but had very little effect on *TLR2* and *NOD1* expression. Upregulation of *TLR4* by IL-1β was significantly reversed by treatment with EcN EVs (*p* < 0.05). In contrast, EcoR12 EVs were unable to counteract the elevated *TLR4* mRNA levels triggered by IL-1β (Figure 3B). In the presence of this pro-inflammatory cytokine, EcN EVs also reduced *NOD1* expression (*p* < 0.05).

### 2.4. Modulation of Pro-Inflammatory and Oxidative Stress Markers by EcN or EcoR12 EVs 

The differential regulation of immune receptors by EVs from the probiotic EcN or the commensal EcoR12 under inflammatory conditions led us to analyze their influence on the expression of inflammatory mediators and oxidative stress markers. This analysis included the cytokines IL-8, TNF-α, and IL-6 and the inflammatory enzymes cyclooxygenase-2 (COX-2) and the inducible nitric oxide synthase (iNOS), due to their pivotal roles in sustaining inflammation in IBD models, as well as the antioxidant enzymes catalase (CAT), superoxide dismutase (SOD), glutathione peroxidase (GPx), and glutathione reductase (GSR), given their critical function in neutralizing free radicals and managing oxidative stress during inflammation and conditions of excessive intestinal serotonin bioavailability. 

**Figure 4:**
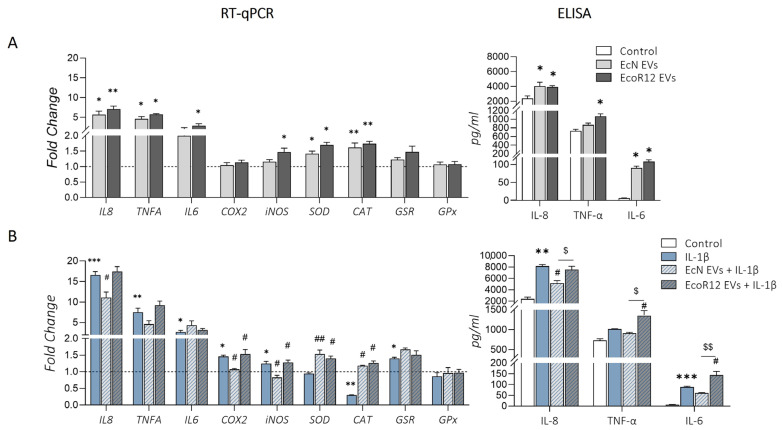
Effects of EcN or EcoR12 EVs on IL-1β-induced production of pro-inflammatory cytokines and oxidative stress enzymes. (**A**) Intact intestinal epithelium model. Caco-2 cells were challenged with EVs (60 µg/mL) from EcN or EcoR12 for 48 h. (**B**) Inflammation model. Caco-2 cells were treated with EVs (60 µg/mL) from EcN or EcoR12 for 3 h and then challenged with IL-1β (10 ng/mL) for 48 h. Relative mRNA levels of the indicated genes were measured through RT-qPCR using GAPDH as the reference gene. Secreted IL-8, IL-6, and TNF-α were quantified through ELISA in the culture supernatants. In all panels, data are expressed as mean ± SEM from three independent experiments. Differences were evaluated with one-way ANOVA, followed by post hoc Tukey’s. * *p* ≤ 0.05, ** *p* ≤ 0.01, *** *p* ≤ 0.001 vs. control cells (white bars or dashed lines); ^#^ *p* ≤ 0.05, ^##^ *p* ≤ 0.01 vs. IL-1β-treated cells; ^$^ *p* ≤ 0.05, ^$$^ *p* ≤ 0.01, significance between cells stimulated with EcN and EcoR12 EVs.

In the model of the intact intestinal epithelium (Figure 4A), EVs from both strains significantly induced *IL8*, *IL6,* and *TNFA* mRNA levels (*p* < 0.05) compared to untreated control cells. The pro-inflammatory cytokines were also analyzed at the protein level through ELISA in the culture supernatants. Consistent with the mRNA profiles, the levels of secreted IL-8 and IL-6 were significantly higher in Caco-2 cells incubated with EcN or EcoR12 EVs than in untreated control cells (*p* ≤ 0.01). Regarding the antioxidant enzymes, both EcN and EcoR12 EVs triggered upregulation of *SOD* (*p* < 0.05) and *CAT* (*p* < 0.01) compared to control cells. Additionally, *iNOS* expression was induced specifically in response to EcoR12 EVs (*p* < 0.05). No significant changes in the mRNA levels of *COX2*, *GSR,* or *GPx* were observed between cells treated with EVs and those not treated with EVs.

In the model of IL-1β-induced inflammation, treatment with this cytokine promoted a significant upregulation of all of the pro-inflammatory cytokines analyzed (Figure 4B). Again, the modulatory effects mediated by EVs from EcN or EcoR12 were strain-specific. In the presence of IL-1β, treatment with EVs from the probiotic EcN significantly reduced *IL8* and *TNFA* mRNA levels (*p* < 0.05). In contrast, EcoR12 EVs were unable to counteract the increased expression of these genes triggered by IL-1β. At the protein level, ELISA assays confirmed the anti-inflammatory effects of EcN EVs assessed according to their ability to diminish the secreted levels of IL-8 and IL-6 cytokines (*p* < 0.05). 

On the contrary, treatment with EcoR12 EVs did not diminish the secretion of any of the pro-inflammatory cytokines analyzed. This resulted in cytokine concentration values that were similar to or even higher than those observed in Caco-2 cells treated with IL-1β alone. Additionally, *COX2* and *iNOS* were significantly upregulated under conditions of IL-1β-induced inflammation in Caco-2 cells (*p* < 0.05). In this context, treatment with EVs EcN prevented the IL-1β-induced upregulation of both genes (*p* < 0.05), resulting in *COX2* and *iNOS* mRNA levels that were similar to those of untreated control cells. In contrast, EcoR12 EVs did not exhibit any compensatory effect on *COX2* and *iNOS* expression (Figure 4B). Regarding the antioxidant enzymes, the results showed a significant downregulation of *CAT* mRNA levels under conditions of IL-1β-induced inflammation (*p* < 0.01 vs. control), which was prevented by treatment with either EcN EVs (*p* < 0.01) or EcoR12 EVs (*p* < 0.05). In contrast, both treatments involving microbiota/probiotic EVs did not counteract the increased expression of GSR under inflammatory conditions. Although IL-1β did not modify *SOD* expression, upregulation of this gene by EcN and EcoR12 EVs was also observed in the presence of this pro-inflammatory cytokine.

### 2.5. Effects of EcN or EcoR12 EVs on IL-1β-Induced Epithelial Barrier Damage

Intestinal inflammation and serotonin dysregulation are linked to alterations in the epithelial barrier’s integrity/permeability, especially affecting TJ-associated proteins [43,44]. In this context, we sought to analyze the ability of EcN EVs or EcoR12 EVs to preserve epithelial barrier integrity in Caco-2 cell monolayers challenged with IL-1β in comparison with normal intestinal epithelium conditions. 

**Figure 5:**
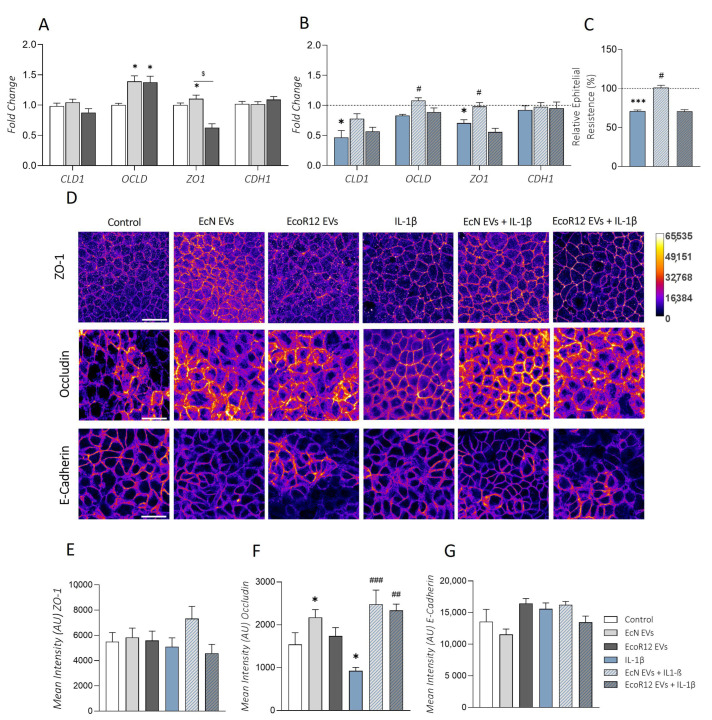
Effects of EcN or EcoR12 EVs on preventing IL-1β-induced alterations on epithelial tight junctions. The bar code for all the conditions analyzed is indicated next to panel G. (**A**) Gene expression analysis of TJ proteins in Caco-2 cell monolayers treated with EVs (60 µg/mL) from EcN or EcoR12 for 48 h (model of intact intestinal epithelium). (**B**) Gene expression analysis of TJ proteins in Caco-2 cell monolayers in the IL-1β-induced inflammation model. In both panels, relative mRNA levels of the indicated genes were measured through RT-qPCR using GAPDH as the reference gene. (**C**) TER measurement under conditions of IL-1β-induced inflammation. TER values were measured before and after 48 h treatment with IL-1β in the presence and absence of EcN EVs or EcoR12 EVs. Data are presented as percentage of changes in TER from the initial value compared to untreated control cells, which were assigned 100% (dashed line). (**D**) Immunofluorescence staining of ZO-1, occludin, and E-cadherin in Caco-2 cell monolayers challenged with the indicated treatments. Representative confocal maximal projection images are shown in Fire LUT after image processing. Calibration bar of Fire LUT intensity is shown on the right. Scale bar: 20 µm. (**E**–**G**) Quantification of ZO-1, occludin, and E-cadherin total mean intensity, respectively, in Caco-2 cells after the indicated treatments. Data are presented as arbitrary intensity units (AU). In all panels, data are expressed as mean ± SEM from three independent experiments. Differences were evaluated with one-way ANOVA, followed by post hoc Tukey’s. * *p* ≤ 0.05, *** *p* ≤ 0.001 vs. control cells (white bars or dashed line depending on the panel); ^#^ *p* ≤ 0.05, ^##^ *p* ≤ 0.01, ^###^ *p* ≤ 0.001 vs. IL-1β-treated cells; ^$^ *p* ≤ 0.05, significance between cells stimulated with EcN and EcoR12 EVs.

The epithelial barrier function was analyzed by measuring the transepithelial electrical resistance (TER). To this end, Caco-2 cell monolayers grown in Transwell membrane supports were challenged with IL-1β for 48 h in the absence or presence of EVs from EcN or EcoR12. Untreated cell monolayers were used as a control. Treatment with IL-1β significantly reduced the TER values of polarized cell monolayers compared to untreated control cells. However, apical stimulation with EcN EVs neutralized the decrease in TER caused by this pro-inflammatory cytokine, with values that did not significantly differ from those of untreated control cells. In contrast, EcoR12 EVs did not prevent IL-1β-mediated epithelial barrier disruption (Figure 5C).

Concerning the impact on tight junctions, the expression of major TJ proteins was analyzed both at the mRNA and protein levels after 48 h treatment. Under conditions of a normal intestinal epithelium, EVs isolated from both strains significantly upregulated occludin (*OCLD*) compared to untreated control cells (*p* ≤ 0.05). In addition, EcN EVs also caused a significant upregulation of *ZO1* (*p* ≤ 0.05). None of the treatments involving EVs altered the mRNA levels of *CLD1* (encoding claudin-1) or *CDH1* (encoding E-cadherin) compared to control cells (Figure 5A). We also carried out immunofluorescence staining followed by confocal laser scanning microscopy of ZO-1, occludin, and E-cadherin (Figure 5D). The results for occludin and E-cadherin were consistent with their gene expression levels (Figure 5F,G). Concerning ZO-1, microscopy images showed an increased signal in the cell boundaries of Caco-2 monolayers treated EcN EVs, whereas this effect was not apparent in cells incubated with EcoR12 EVs. In this case, the ZO-1 signal was similar to that of control cells (Figure 5D). The upregulation and peripheral distribution of ZO-1 by EcN EVs in Caco-2 cell monolayers was previously reported by our group [30]. However, the increased expression of occludin after 48 h of incubation with either EcN or EcoR12 EVs was been observed at 24 h [30].

Under conditions of IL-1β-induced inflammation, exposure of Caco-2 cells to this pro-inflammatory cytokine significantly affected the expression of TJ proteins. Specifically, this treatment led to a significant downregulation of *CLD1* and *ZO1* compared to untreated control cells (*p* ≤ 0.05), with a trend towards reduced OCLD expression (Figure 5B). In contrast, expression of *CDH1* was not affected by IL-1β treatment. Importantly, at the gene expression level, only EcN EVs were able to counteract the negative impact of IL-1β on *ZO1* and *OCLD* mRNA levels (*p* < 0.05), yielding values that were similar to those of untreated control cells. Clearly, EcoR12 EVs did not prevent downregulation of *ZO1* in cells treated with IL-1β but tended to increase the *OCLD* expression levels, although differences did not reach statistical significance (Figure 5B). The upregulation of *ZO1* by EcN EVs was accompanied by the redistribution of the protein to the cell boundaries, as evidenced by the immunofluorescence signal (Figure 5D,E). Although the occludin protein levels were higher in the presence of either EcN or EcoR12 EVs compared to IL-1β-treated cells, differences in the subcellular distribution were apparent depending on the strain. In particular, there was a lower peripheral occludin signal in Caco-2 cells incubated with EcoR12 EVs (Figure 5D,E).

## 3. Discussion

IBD is a chronic, relapsing, and multifactorial inflammatory condition that involves environmental factors, alterations in local and systemic immune responses, and imbalances in the gut microbiota composition in genetically susceptible individuals. The two main forms of IBD are CD and UC, which differ in the location and nature of inflammation. While CD can cause transmural inflammation and affect any part of the gastrointestinal tract, UC inflammation is limited to the colonic mucosa [45,46]. Several genetic variants have been associated with disease susceptibility, with most affecting barrier- and immune-related genes. This could contribute to the exacerbated immune response [47]. Regarding the factors triggering the initiation and pathogenesis of chronic IBD, accumulating evidence implicates altered host defense responses against the gut microbiota at the mucosal interface, resulting in disrupted intestinal epithelial barrier function [48]. 

Serotonin is an important mediator of intestinal homeostasis, and it functions under physiological and pathological conditions. Disturbances in serotonin bioavailability and signaling have been linked to the onset and severity of gut inflammatory disorders, particularly in IBD [10,18,19]. Both the gut microbiota and inflammation mediators modulate the expression of genes involved in serotonin synthesis, reuptake, and signaling. In turn, serotonin influences the integrity/permeability of the epithelial barrier, intestinal motility and secretion, inflammatory responses, and the gut microbiota balance [23]. In IBD, elevated serotonin levels and/or signaling exacerbate the inflammatory state and dysbiosis. The strong connection between the gut microbiota, barrier function, and immunity has encouraged the use of probiotics as a promising therapeutic strategy to ameliorate inflammation and restore intestinal homeostasis in IBD [49]. In this context, clinical trials have revealed the therapeutic benefits of the probiotic EcN in inducing and maintaining the remission of UC [50,51]. 

Under physiological conditions, the regulation of serotonin metabolism by the gut microbiota has primarily been studied in cellular models and germ-free animals [10]. The microbial factors involved in such regulation include secreted bacterial metabolites [8,52] and bacterial EVs [12,13]. However, whether these secreted microbial factors could attenuate disturbances in the serotonin system under inflammatory conditions remains a relatively unexplored issue.

Previous studies from our group proved the ability of EVs from probiotic and commensal *E. coli* strains to differentially modulate host immune and defense responses to safeguard intestinal homeostasis in several cellular and experimental models [4]. In the present study, we analyzed the ability of EV isolated from the probiotic EcN and the commensal EcoR12 to modulate the serotonin system and related genes in Caco-2 cells under conditions of health and disease affecting the epithelial barrier, including (i) a normal intestinal epithelium model and (ii) an IL-1β-induced inflammation model. We have applied this inflammation model because IL-1β is an important mediator of inflammation and tissue damage in IBD [39,53]. Elevated levels of IL-1β are correlated with disease severity. This pro-inflammatory cytokine induces barrier dysfunction by disrupting TJs [43], leading to increased intestinal permeability, which exacerbates dysregulated inflammatory responses in IBD patients. Indeed, immune blockade of IL-1β has shown therapeutic effects in experimental colitis mouse models [54]. 

The results from the present study showed that in the absence of IL-1β challenge, EVs from both strains elicit similar regulatory effects, principally affecting serotonin bioavailability and the expression of the antioxidant enzymes CAT and SOD. Cells exposed to EcN or EcoR12 EVs secreted higher serotonin levels than control cells. At the gene level, both types of EVs triggered upregulation of SERT mRNA and protein levels (clearance pathway), but only EcN EVs were able to activate the expression of the rate-limiting enzyme of the serotonin biosynthesis pathway TPH1. These findings suggest that under physiological conditions, EVs from these microbiota *E. coli* strains may help to fine-tune appropriate intestinal serotonin levels by regulating genes involved in the serotonin circuit. It should be noted that maintaining a proper level of free intestinal serotonin is essential for preserving gut function through interactions with specific receptor subtypes.

The observed effects on serotonin genes are consistent with those reported for the EVs of beneficial microbiota species, such as *A. muciniphila* and *F. prausnitzi,* in the Caco-2 model of the intestinal epithelium or in healthy mice [12,13]. The ability of live suspensions of the probiotic EcN to enhance the bioavailability of serotonin in gut tissue explants through modulation of the synthesis and clearance pathways has already been described, although the nature of the effector signal was unknown [11]. Here, we show that the effects are mediated by released EVs. In addition, our results show that EcN and EcoR12 EVs can improve the intestinal barrier function by upregulating *AHR*. It is known that *E. coli* strains metabolize dietary tryptophan into indole-related compounds, which exert beneficial effects on the integrity and function of the intestinal mucosa through their interaction with the AHR nuclear receptor [55,56]. Therefore, the positive regulation of AHR expression by EVs released by intestinal *E. coli* strains provides a mechanism to reinforce the action of microbial indole metabolites in preserving intestinal homeostasis and microbiota balance.

Under inflammatory conditions, the effects elicited by the EVs differed depending on whether they were produced by the probiotic EcN or the commensal EcoR12. Concerning the regulation of the serotonin system, only EcN EVs were able to prevent the increased production of serotonin induced by IL-1β treatment. In the context of serotonin bioavailability, SERT has a key role in controlling free serotonin levels in the intestinal mucosa by promoting its reuptake by enterocytes. Continuous clearance is needed for the proper functioning of the serotonin circuit to regulate the biological effects of released serotonin. Indeed, reduced expression of SERT has been observed in the epithelium of CD and UC patients [20]. Our results also showed reduced SERT mRNA and protein levels in Caco-2 cells after IL-1β treatment. Interestingly, both EcN and EcoR12 EVs prevented SERT downregulation, resulting in mRNA and protein levels that were higher than those of untreated control cells. The fold-change increase in SERT expression (mRNA and protein) induced by EcN or EcoR12 EVs was comparable between cells treated under normal and inflammatory conditions, suggesting that the regulatory mechanism was not altered by inflammation mediators. In contrast, *TPH1* induction by EcN EVs in normal Caco-2 cells was abolished in the presence of IL-1β. This fact may explain, at least in part, the reduced levels of secreted serotonin in cells treated with EcN following the inflammation model. In addition, SERT overexpression should also correlate with reduced levels of free serotonin. The results obtained with EcN EVs matched this hypothesis. However, in cells treated with IL-1β in the presence of EcoR12 EVs, extracellular serotonin did not diminish (vs. IL-1β) despite elevated SERT levels. This discrepancy may have several explanations, such as the involvement of other regulatory mechanisms affecting SERT activity and/or membrane trafficking, as well as subcellular distribution. In intestinal epithelial cells, serotonin reuptake activity relies on the proper location of SERT in the apical membrane [15]. Moreover, diverse post-translational modifications of the serotonin transporter have been studied, with a particular focus on their influence in the brain’s serotonin neurotransmitter function [57,58]. In the gut, there is evidence that SERT is post-transcriptionally regulated by miR-24 and miR-200a. Both miRNAs were found upregulated in intestinal mucosa samples from both IBD patients and experimental IBD animal models [41,42,59]. The elevated expression of these miRNAs in IBD correlates with reduced SERT mRNA and protein levels in gut tissue samples. In addition, high levels of miR-24 in UC have been associated with impaired barrier integrity due to the downregulation of the TJ protein cingulin [22]. The results from the present study show that EVs from the probiotic EcN prevented the increase in miR-24 and miR-200a levels induced by IL-1β in Caco2 cells. This regulatory effect may explain the counteracting activity of EcN EVs on SERT expression and free serotonin under inflammatory conditions. In contrast, miR-24 and miR-200a expression did not differ between cells challenged with IL-1β in the absence and presence of EVs from the commensal EcoR12. It is important to note that the regulation of SERT involves intricate molecular interactions that encompass miRNAs, transcription factors, and signaling pathways. Thus, miR-24 and miR-200a are merely a couple of contributors to this complex network. In this context, we focused our attention on studying the influence of EcN and EcoR12 EVs on the expression of TLR2, TLR4, and NOD1, immune receptors activated by microbiota molecules and known to influence intestinal serotonin bioavailability by downregulating SERT expression and function [15,60]. In addition, overexpression of TLR2, TLR4, and their coreceptor CD14 has been found in different sections of the intestinal mucosa of UC and CD patients depending on the disease stage [61]. Our results showed that the IL-1β-driven inflammation in Caco-2 cells resulted in higher TLR4 mRNA levels, which could be significantly reduced by EcN EVs but not by EcoR12 EVs. In the inflammation model, treatment with EcN EVs also diminished NOD1 expression. Consistently, in cells treated with IL-1β, the elevated expression and secretion of pro-inflammatory mediators that are modulated by TLR downstream signaling pathways, such as IL-8, TNF-α, COX-2, and iNOS, could only be counteracted by EVs from the probiotic strain. The lack of correlation between SERT and its regulators TLR4, miR-24, and miR-200a in cells treated with EcoR12 EVs suggests the involvement of other regulatory mechanisms that have not yet been identified.

Overall, the results suggested that EVs from the probiotic EcN may preserve SERT expression under conditions of intestinal inflammation through several regulatory mechanisms, including the downregulation of the microRNAs miR-24 and miR-200a and the immune receptors TLR4 and NOD1. To our knowledge, this is the first study demonstrating the regulation of these miRNAs by probiotic-derived EVs. Considering that microbiota EVs carry a wide diversity of cargo molecules, including common MAMPs that are recognized by immune receptors, strain-specific produced molecules should mediate the differential EVs’ effects.

Oxidative stress is tightly associated with inflammatory responses and, therefore, it has been implicated in the development and exacerbation of IBD [62]. In this context, intestinal inflammatory mediators, including serotonin, have been shown to inhibit the expression of antioxidant enzymes in intestinal epithelial cells [60]. It has been proposed that this prooxidant mechanism might help to exacerbate inflammation by damaging the intestinal epithelium. In our inflammation model, the ability of IL-1β to promote oxidative stress was evidenced by two regulatory findings: (i) upregulation of the inflammatory enzymes COX-2 and iNOS involved in the generation of reactive oxygen/nitrogen species, and (ii) downregulation of the antioxidant enzyme CAT. Importantly, EcN EVs performed better than EcoR12 EVs in counteracting the IL-1β-induced alterations. Whereas EcN EVs prevented changes in the expression of both prooxidant and antioxidant enzymes, EcoR12 EVs only positively affected the antioxidant enzymes.

Disruption of the intestinal epithelial barrier and increased intestinal permeability are well-recognized hallmarks of mucosal inflammation that contribute to the severity of IBD [63,64]. Moreover, in inflamed intestinal tissue, dysregulation of serotonin levels can aggravate intestinal inflammation, motility, and diarrhea [44,65]. The permeability of the epithelial barrier is controlled by epithelial TJs. Previous results from our group proved the ability of EcN EVs to strengthen the intestinal epithelial barrier and reduce gut permeability by modulating the TJ proteins ZO-1, claudin-14, and claudin-2 in Caco-2 cell monolayers [30]. Additionally, they protect the barrier’s integrity against damage caused by enteropathogenic *E. coli* by counteracting the altered expression and subcellular distribution of TJ proteins [66]. The results from the present study show that EVs from the probiotic EcN can also prevent IL-1β-induced damage to the epithelial barrier, assessed using TER assays and TJ protein/mRNA expression analysis (ZO-1 and occludin). Again, the effects were strain-specific, as EVs from the commensal EcoR12 could not compensate for the reduced expression of these TJ proteins. 

Current IBD therapies used to control symptoms, such as aminoacylates, corticosteroids, and immunosuppressants, have considerable side-effects. In addition, a significant proportion of patients do not respond to these drugs (non-primer responders) or are prone to losing their response after long treatments [67]. Therefore, there is a need for the discovery of novel effective therapeutic approaches. Among them, microbiome-based strategies are changing the perspective of IBD therapy. The biotic therapies, such as probiotics, prebiotics, and synbiotics, aim to restore the gut microbiota balance in favor of anti-inflammatory and mucosa-healing profiles [24,26,68,69]. The newest component of the “biotics” strategy refers to postbiotics, a term that encompasses bioactive compounds (metabolites, bacterial components, secreted factors) produced by probiotics or gut-beneficial bacteria. In this context, microbiota/probiotic-derived EVs are emerging as future postbiotics as they exhibit the requirements and benefits necessary to fit within this biotherapy group [28,29,70,71]. Due to their nano-size structure and stability and the great variety of cargo molecules, microbiota-derived EVs can penetrate the mucus layer and modulate host intestinal immune and barrier responses. In addition, EVs from gut microbes can cross the epithelial barrier, enter the bloodstream, and reach distal tissues. 

We previously reported that EVs from the probiotic EcN could serve as a safe postbiotic alternative to the utilization of live probiotic bacteria in ameliorating DSS-induced colitis in mice [36], as well as in improving diarrhea, clinical symptoms, and immunity against rotavirus infection in neonatal rats [72]. Results from the present study show that in intestinal epithelial cells, EVs from the probiotic strain EcN attenuate the IL-1β-induced alterations affecting serotonin metabolism, oxidative stress, and barrier integrity, thereby pointing to their potential application as a postbiotic in IBD. Importantly, they provide evidence of the molecular mechanisms underlying the vesicle-mediated reduction of free serotonin levels under inflammatory conditions through the regulation of SERT, miR-200a, and miR-24. Understanding the molecular mechanisms activated by microbiota/probiotic bioactive molecules is essential before their translation to human health as pharmabiotics or functional food ingredients. 

## 4. Materials and Methods

### 4.1. Bacterial Strains and Isolation of EVs

The probiotic *E. coli* Nissle 1917 (EcN) was from Ardeypharm (GmbH, Herdecke, Germany). The *E. coli* strain EcoR12, included in the ECOR reference collection, was isolated from a fecal sample of a healthy human adult [73].

For the isolation of the EVs, bacterial strains were cultured in Luria–Bertani broth for 16 h. Subsequently, bacterial cells were pelleted through centrifugation, and the culture supernatants were used for EV extraction, as described previously [34]. In brief, the supernatants underwent filtration through a 0.22 μm pore size filter (Merck, Millipore, MA, USA) to remove residual bacterial cells. The filtrate was then concentrated using Centricon Plus-70 centrifugal filters with a 100 kDa cutoff (Merck, Millipore, MA, USA). Following an additional filtration step (0.22 μm pore size), vesicles were isolated through ultracentrifugation at 150,000× *g* for 1 h at 4 °C. The pelleted EVs were washed and resuspended in phosphate-buffered saline (PBS). Quantification of the EV samples, in terms of total protein content, was performed using the Pierce BCA method, and sterility was confirmed by plating on LB agar. Aliquots of the EVs were stored at −20 °C until use. Image analysis of EcN and EcoR12 EVs was previously assessed through Cryo-Transmission Electron Microscopy [33].

### 4.2. Cell Culture and Stimulation Conditions

Human colonic epithelial Caco-2 cells (ATCC HTB37) were routinely grown in Dulbecco’s modified essential medium (DMEM) supplemented with 10% fetal bovine serum, 25 mM of HEPES, 1% non-essential amino acids, and 1× penicillin–streptomycin (Corning, Fisher Scientific Inc., Barcelona, SpainProduct Number 30-002-CI). The cultures were maintained at 37 °C in a humidified incubator with 5% CO_2_. The cells were sub-cultured with trypsin-EDTA (Gibco-BRL, Fisher Scientific Inc., Barcelona, Spain) once a week.

The in vitro model of intestinal inflammation used in this study involved Caco-2 cell monolayers exposed to IL-1β (Recombinant Human IL-1 beta/IL-1F2 Protein; Bio-Techne R&D Systems, S.L.U., Madrid, Spain). Caco-2 cells were seeded at a density of 2 × 10^5^ cells/mL in 12-well plates and cultured for 14 days, while replacing the culture medium every 2 days. The cell monolayers were pre-incubated with EcN EVs or EcoR12 EVs at a dose of 60 μg/mL for 3 h and subsequently treated with IL-1β (10 ng/mL). Unless otherwise indicated, the duration of IL-1β treatment was 48 h. Throughout the experiment, both the EVs and IL-1β were maintained in the culture medium. As a control, cells treated with IL-1β in the absence of bacterial EVs were cultured in parallel. For comparison, the effect of EcN or EcoR12 EVs (60 μg/mL) was also analyzed in Caco-2 cell monolayers in the absence of IL-1β-induced inflammation after 48 h of incubation as a model that mimics the normal intestinal epithelium state. In all cases, culture supernatants and cells were collected for ELISA and quantitative RT-PCR assays, respectively.

### 4.3. Cell Viability Assays

To assess the cytotoxic effect of IL-1β on Caco-2 cells, cell viability was determined using the MTT (3-(4,5-Dimethylthiazol-2-yl)-2,5-diphenyl tetrazolium bromide) assay. Each well of a 96-well plate was seeded with 100 μL of Caco-2 cells (1 × 10^5^ cells/mL) and incubated for 24 h at 37 °C. Subsequently, IL-1β was added at concentrations ranging from 1 to 100 ng/mL. Following a 48 h incubation period, cells were treated with a 0.25% MTT solution (Sigma-Aldrich Chemical Co, St. Louis, MO, USA) in PBS and allowed to react for 2 h at 37 °C. The medium was then removed, and 0.1 mL of a solubilization reagent (99% dimethyl sulfoxide) was added. Cell viability was measured at 570 nm using a Variouskan LUX multimode microplate reader (Thermo Fisher Scientific Inc., Barcelona, Spain). The results were expressed as the percentage of cell survival relative to untreated control cells.

### 4.4. Measurement of Transepithelial Electrical Resistance (TER)

Caco-2 cells were seeded at a density of 1 × 10^5^ cells/cm^2^ in the apical compartment of 12 mm polycarbonate Transwell cell culture inserts (0.4 μm, Transwell Millipore, Merck Life Sciences, Madrid, Spain) and cultured for 14 days. The basolateral compartment was filled with 1.5 mL of culture medium. Throughout the growth and differentiation period, the medium in both compartments was replaced every 2 days. The integrity of the cell monolayer was monitored by measuring TER. Stimulations were carried out on Caco-2 cell monolayers with initial TER values exceeding 700 Ω·cm^2^. Apical stimulation was performed with EcN EVs or EcoR12 EVs (60 μg/mL) for 3 h, followed by IL-1β treatment. After 48 h, the Caco-2 cell monolayers were washed in PBS, and TER was evaluated with a Millicel-ERS-2 voltmeter (Millipore, Merck Life Sciences, Madrid, Spain). The ohmic resistance of a blank (cell-free culture inset) was also measured. To obtain the sample resistance, the blank value was subtracted from the total sample resistance. The final unit area resistance (Ω cm^2^) was calculated by multiplying the sample resistance (Ω) by the effective membrane area (1.12 cm^2^).

### 4.5. RNA Extraction and Quantitative Reverse Transcription–Polymerase Chain Reaction (RT-qPCR)

Gene expression was analyzed through RT-qPCR. The total RNA was extracted using the miRNeasy mini Kit (Qiagen, Crawley, UK) according to the manufacturer’s protocol. First, 1 µg of RNA was reverse-transcribed in a final volume of 20 µL using the High-Capacity cDNA Reverse Transcription kit (Applied Biosystems, Foster City, CA, USA). Quantitative PCR reactions were carried out on a QuantstudioTM^3^ real-time PCR system (Applied Biosystems) utilizing SYBR^®^ Green PCR Master Mix (Applied Biosystems) using specific oligonucleotides for genes related to the serotonergic pathway (*SERT*, *TPH1*, *MAO*, *AHR*), inflammation (*IL6*, *IL8*, *TNFA*, *TLR2*, *TLR4*, *NOD1*), tight junctions (*CDH1*, *ZO1*, *OCLDN*, *CLDN1*), and oxidative stress (*SOD*, *CAT*, *GSR*, *GPx*, *COX2*, *iNOS*), which are listed in Table S1. The housekeeping *GAPDH* gene was used as the internal control. The reaction program included one denaturation cycle for 10 min at 95 °C, followed by 40 cycles of 15 s at 95 °C and 1 min at 60 °C. A control reaction was carried out in the absence of RNA.

For microRNA expression analysis, reverse transcription of RNA (5 ng/μL) was performed using the miRCURY LNA RT kit (Qiagen, Iberia S.L., Barcelona, Spain), followed by quantitative PCR using the miRCURY LNA PCR Assay (Qiagen). The U6 snRNA reference gene was used for normalization. The primers used were hsa-miR-24-3p miRCURY LNA primer (Qiagen) (MiRBase accession # MIMAT0000080), hsa-miR-200-a3p miRCURY LNA primer (Qiagen) (MiRBase accession # MIMAT0000682), and the U6 snRNA control primer set (Qiagen). The standard PCR program consisted of one denaturation cycle for 2 min at 95 °C, followed by 40 cycles of 10 s at 95 °C and 1 min at 56 °C.

For both mRNA and miRNA analyses, the relative gene expression was calculated using the 2^−ΔΔCt^ formula. The data were presented as the fold change relative to the experimental control (untreated cells or IL-1β-treated cells, depending on the model).

### 4.6. Quantification of Cytokines and Serotonin (5-HT) through ELISA 

For these assays, a total of 2 × 10^5^ Caco-2 cells were initially seeded in 12-well plates and cultured for a duration of 14 days. Following the indicated treatments, the culture supernatants were collected, centrifuged at 10,000× *g* for 20 min at 4 °C, and stored at −80 °C until analysis. The secreted levels of IL-8, IL-6, and TNF-α were quantified using enzyme-linked immunosorbent assay (ELISA) sets (BD Biosciences, San Jose, CA, USA) according to the manufacturer’s instructions. The results were expressed as pg/mL. The serotonin concentration was assessed using the 5-HT (Serotonin/5-Hydroxytryptamine) ELISA Kit supplied by Elabscience Biotechnology Co., Ltd., Tebu-Bio S.L., Barcelona, Spain in accordance with the manufacturer’s guidelines. The results were expressed in ng/mL.

### 4.7. Immunofluorescence Labelling

Caco-2 cells were seeded at a density of 1 × 10^4^ cells/mL in an 8-well chamber slide (µ-Slide 8 well Glass bottom, Ibidi, Inycom, Zaragoza, Spain). Cells were grown for 7 days and then stimulated as described above. At the end of the incubation period, cells were washed with PBS, fixed with 4% paraformaldehyde in PBS, permeabilized with 0.05% saponin (Sigma-Aldrich, Chemical Co., St. Louis, MO, USA), and blocked using PBS containing 1% bovine serum albumin, as previously described [31]. The TJ proteins ZO-1, E-cadherin, and occludin were stained using, respectively, anti-ZO-1 (1:100, Invitrogen, Fisher Scientific Inc., Barcelona, Spain, Catalog # 33-9100), anti-E-cadherin (1:70, BD Biosciences, Catalog # 610181), and anti-occludin (1:1000, Invitrogen, Catalog # 331588) mouse IgG monoclonal antibodies for 2 h at room temperature, followed by incubation with Alexa-Fluor-488-conjugated F(ab’)2 goat anti-mouse IgG (H+L) polyclonal antibody (1:500, Invitrogen, Catalog #A-11029) for 3 h at room temperature. Nuclei were labeled with DAPI (0.125 µg/mL, Sigma Aldrich, Chemical Co., St. Louis, MO, USA) for 20 min at room temperature. Immunostaining of SERT was performed using anti-SERT Recombinant Rabbit Polyclonal Antibody (1:250, Thermo Fisher, Catalog # 711108,) for 3 h at room temperature and Goat anti-Rabbit IgG (H+L) Superclonal™ Secondary Antibody, Alexa Fluor^®^ 488 conjugate (1:500, Thermo Fisher, Catalog # A27034) for 2 h at room temperature. Immunolabelling of ZO-1 and occludin was previously established in our laboratory [31]. For SERT and E-cadherin immunostaining, antibody negative control assays were performed to rule out nonspecific binding of the secondary antibodies. 

### 4.8. Confocal Microscopy

Cells were observed using a Zeiss LSM880 confocal microscope (Carl Zeiss Iberia S.L., Jena, Germany) equipped with a 63×/1.4 objective, an Argon laser, and a 405 nm laser diode. The 488 nm line of the Argon laser was used to collect the fluorescence of the Alexa Fluor 488, and the 405 nm diode was used to detect the DAPI emission fluorescence. Images were acquired with a voxel size of 0.13 µm × 0.13 µm × 0.37 µm (x, y, z, respectively). For each experimental condition and replicates (*n* = 3), 5 different fields of view with 40–100 cells each were acquired.

### 4.9. Image Analysis

Images were processed and analyzed using the Fiji software v1.53t [74]. The mean intensity measurement of ZO-1 staining was performed following the protocol described previously [30]. Mainly, images were filtered and processed to remove the background and to finally trace the tight junction fluorescence using the Tubeness plugin (sigma = 0.13) and project using the maximum intensity projection method. Segmentation was performed by using a pre-trained Labkit classifier [75].

To analyze the mean intensity of SERT staining, images were first filtered with a median filter (radius = 1) and then segmented using the Huang algorithm [76]. Measurements were performed on all focal planes of the original images masked with the previously obtained binary stacks. 

### 4.10. Statistical Analysis

The data were collected from a minimum of three independent biological experiments, each conducted in triplicate. Statistical analysis and graph generation were performed using GraphPad Prism 7.0 software (GraphPad Software, Inc., La Jolla, CA, USA). Data were tested for normal distribution using the Shapiro–Wilk test. All data are presented as the mean ± standard error (SEM). Group comparisons were assessed through one-way analysis of variance (ANOVA) followed by Tukey’s post-test. A *p*-value of <0.05 was considered statistically significant.

## 5. Conclusions

This study provides new insights into the regulation of serotonin metabolism and related intestinal functions by EVs of probiotic and commensal *E. coli* strains in intestinal epithelial cells, with a focus on their potential use as a postbiotic strategy for treating IBD. Considering that intestinal serotonin levels and microbiota EVs are important mediators in the gut–brain communication axis, the results presented here point to other potential applications of microbiota EVs in treating neurological disorders associated with leaky gut. Further research and clinical trials are needed to confirm the benefits of microbiota EV-based interventions in leaky gut and serotonin-related disorders in humans.

## Data Availability

The original contributions presented in this study are included in the article/Supplementary Materials. Further inquiries can be directed to the corresponding author.

## Supplementary Materials

The following supporting information can be downloaded at: https://www.mdpi.com/article/10.3390/ijms25105338/s1.
