# Supplementary material for: Modulation of Serotonin-Related Genes by Extracellular Vesicles of the Probiotic Escherichia coli Nissle 1917 in the Interleukin-1β-Induced Inflammation Model of Intestinal Epithelial Cells
Source: Int J Mol Sci. 2024 May 14;25(10):5338. doi: 10.3390/ijms25105338 (PMC11121267; doi:10.3390/ijms25105338)
Supplement: Supplementary file 1 [file ijms-25-05338-s001.zip › ijms-3007859-supplementary.pdf]

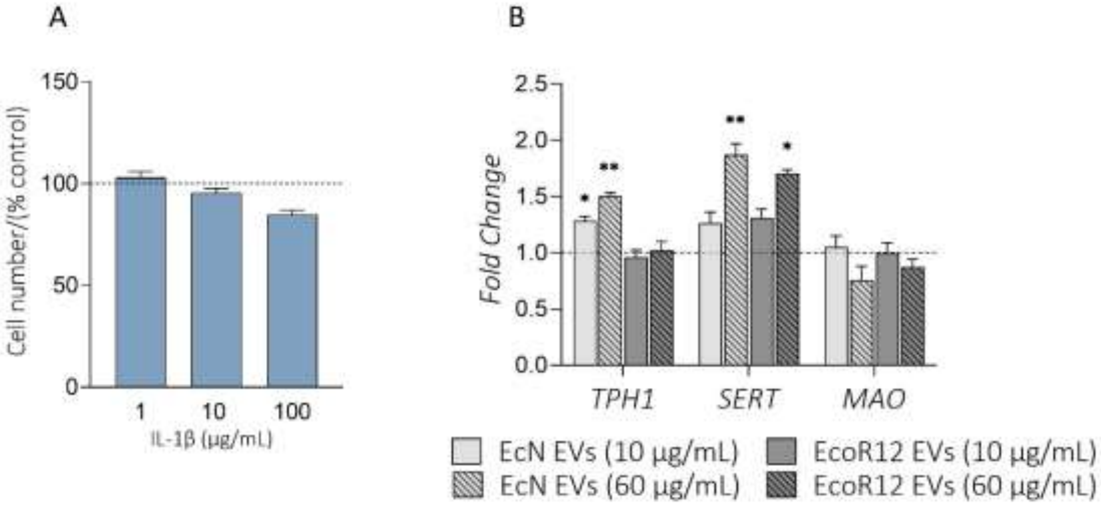

Figure S1. Setting up the experimental model. (A) Effect of the IL-1β concentration on cell viability assessed by the MTT assay. Caco-2 cell monolayers were exposed to the indicated of IL-1β for 48 hours. Untreated control cells were incubated in parallel (dashed line). **(B)** Influence of the EV concentration on the expression of the serotonergic genes *TPH-1, SERT,* and *MAO*. Caco-2 cells were exposed to EVs from the probiotic EcN or the commensal EcoR12 (10 and 60 µg/mL) for 48 hours. Untreated cells were incubated in parallel as a control (dashed line). The relative mRNA levels of the indicated genes were measured by RT-qPCR using GAPDH as the reference gene. In all panels, data are expressed as mean ± SEM from three independent experiments. Differences were evaluated with one-way ANOVA, followed by post hoc Tukey's. * $p \leq 0.05$, ** $p \leq 0.01$ *vs* control.

Supplementary Table S1. Sequences of primers used for quantitative RT-PCR.

Gene	*Forward Sequence (5′-3′)*	*Reverse Sequence (5′-3′)*
SERT	TTCAACAACAACTGCTACCA	ACACATCTTCATTCCTCATCTC
TPH1	TGCAAAGGAGAAGATGAGAGAATTTAC	CTGGTTATGCTCTTGGTGTCTTTC
MAO	TTCAGGACTATCTGCTGCCAA	GGTCCCACATAAGCTCCACC
AHR	CAAATCCTTCCAAGCGGCATA	CGCTGACCTAAGAACTGAAAG
IL6	CACAGATGAAGGTGGGAAGGATG	TGCTAAGCAAACAGGCACGACTA
IL8	CTGGCCGTGGCTCTCTTG	GGGTGGAAAGGTTTGGAGTATG
TNFA	AACTAGTGGTGCCAGCCGAT	CTTCACAGAGCAATGACTCC
TLR2	GAAAGCTCCCAGCAGGAACATC	GAATGAAGTCCCGCTTATGAAGACA
TLR4	TTGAGCAGGTCTAGGGTGATTGAA	ATGCGGGACACACACACTTTCAAATA
NOD1	GTACGTCACCAAAATCCTGGA	CAGTCCCCTTAGCTGTGATC
CDH1	GAAGGTGACAGAGCCTCTGGAT	GATCGGTTACCGTGATCAAAAT
ZO1	TGAGGCAGCTCACATAATGC	GGTCTCTGCTGGCTTGTTTC
OCLD	TTTGTGGGACAAGGAACACA	TCATTCACTTTGCCATTGGAT
CLD1	GCCCCAGTGGAGGATTTACT	GTTTTGGATAGGGCCTTGGT
SOD	ATCCTCTATCCAGAAAACACG	ACACCACAAGCCAAACGAC
CAT	TGTTGAAGATGCGGCGAG	ATGAGAGGGTAGTCCTTGTG
GSR	GATCCCAAGCCCACAATAGA	CTTAGAACCCAGGGCTGACA
GPX	GCCTTCCCGTGTAACCAGT	GCGAACTCTTTGATCTCTTCGT
COX2	GGGTTGCTGGGGGAAGAAATG	GGTGGCTGTTTTGGTAGGCTG
iNOS	GTTGAAGACTGAGACTCTGG	ACTAGGCTACTCCGTGGA
GAPDH	GAGTCAACGGATTTGGTCGT	GACAAGCTTCCCGTTCTCAG
